# Pyroptosis in Kawasaki disease: from mechanisms to targeted interventions

**DOI:** 10.3389/fimmu.2025.1566985

**Published:** 2025-04-16

**Authors:** Xiang-Yu Han, Hui-Ru Qi

**Affiliations:** ^1^ Renal Division, Department of Medicine, Peking University First Hospital, Peking University Institute of Nephrology, Beijing, China; ^2^ Capital Institute of Pediatrics, Peking University Teaching Hospital, Beijing, China

**Keywords:** Kawasaki disease, pyroptosis, inflammation, mechanism, intervention

## Abstract

Kawasaki disease (KD) is a relatively common autoimmune disease of childhood, characterized by systemic vasculitis and involvement of the cardiovascular system, particularly the coronary artery. Progressive inflammatory cascades and vascular injury are regarded as two major processes underlying KD. Although it is regarded as a self-limiting disease, some children exhibit resistance to intravenous immunoglobulin (IVIG) treatment, which can lead to the development of life-threatening coronary artery aneurysms that persist into adulthood. Pyroptosis, a special inflammatory cell death pattern, results in the intense release of inflammatory mediators and injuries of tissues such as endothelial cell damage. Evidence from *in vitro* studies and animal models suggests that pyroptosis and associated inflammatory cascades may play a significant role in KD. Here, we highlight the latest insights into pyroptosis in KD and explore the potential therapeutic interventions that target pyroptosis.

## Introduction

Kawasaki disease (KD) was first reported by Japanese pediatrician, Dr. Tomisaku Kawasaki in 1967, and is also referred to as mucocutaneous lymph node syndrome (1, 2). It is distinguished by a set of defining clinical features, including persistent fever, conjunctival congestion, a distinctive strawberry-like tongue, a characteristic rash, changes in the peripheral limbs, and non-suppurative cervical lymphadenopathy (3) (**Table 1**). It is recognized as the leading cause of acquired heart disease in children, particularly those under the age of 5 years old (4). The incidence rate of KD exhibits variability across different racial groups and geographical locations. Notably, in certain areas with higher prevalence, such as Japan, the incidence rate among children under the age of 5 is particularly significant, reaching as high as 1 in 100 (5). Since the onset of the severe acute respiratory syndrome coronavirus type 2 (SARS-CoV-2) pandemic, the epidemiological landscape of Kawasaki disease has experienced substantial shifts across various regions. Amidst the rigorous implementation of epidemic prevention strategies, there has been a marked reduction in the incidence of Kawasaki disease, with decreases ranging from approximately 20% to 40% (6, 7). Despite its classification as an autoimmune disease, no conventional autoantigen has been found until now in KD, likely due to the complexity of the inflammatory cascades involved. The application of intravenous immunoglobulin (IVIG) has dramatically improved the outcome of patients with KD, however, a notable proportion of patients (4%) still progress to develop coronary artery aneurysms (8, 9).

**Table 1:** Clinical findings and diagnostic criteria for KD.

Category	Clinical findings/diagnostic criteria
Common clinical manifestations and major criteria (2, 3)	
Fever	Persistent fever for more than 5 days, often exceeding 39°C
Rash	Polymorphic skin rash
Conjunctivitis	Bilateral, non-exudative conjunctivitis
Oral mucosal changes	Pharyngeal erythema, strawberry tongue, oral mucosal erythema
Limb changes	Acute: swelling of hands and feet; convalescent: periungual desquamation
Lymphadenopathy	Cervical lymph node ≥1.5 cm in diameter
Diagnostic criteria	
Minor criteria	
BCG scar erythema	Erythema at the site of BCG vaccination
Cardiac involvement	ECG abnormalities, echocardiographic evidence of coronary artery dilatation, or other cardiac anomalies
Laboratory findings	Elevated WBC count, elevated CRP, increased ESR
Diagnostic conditions	
Confirmed diagnosis	Fulfillment of five major criteria, or four major criteria plus evidence of coronary artery abnormalities
Suspected diagnosis	Fulfillment of three major criteria, or two major criteria plus two minor criteria

BCG, Bacillus Calmette–Guérin; WBC, white blood cell, CRP, C-reactive protein; ESR, erythrocyte sedimentation rate.

Nevertheless, a limited understanding of the pathophysiology of KD has impeded the identification of the etiological events that lead to vasculitis and cardiovascular involvement. Inflammation and endothelial cell injury are two critical processes in the underlying pathological mechanisms (10). The infiltration of immune cells, particularly in the coronary arteries, is a common feature during the acute phase of KD (11, 12). Monocytes/macrophages and associated pro-inflammatory cytokines, such as interleukin-1 beta (IL-1β), play a significant role in vascular endothelial damage. Evidence from both *in vitro* and *in vivo* studies underscore the central role of IL-1β in KD and its related complications, strongly suggesting the involvement of pyroptosis in the pathological process of KD (10, 13, 14).

Recent findings have highlighted that children from certain ethnic backgrounds are displaying the characteristic symptoms of KD, such as strawberry tongue, rash, and conjunctival congestion, subsequent to exposure to SARS-CoV-2 and *Yersinia pestis* (15, 16). These microorganisms trigger a swift cell-damaging process known as pyroptosis, characterized by the activation of the inflammasome and the subsequent action of the gasdermin family of proteins, which function as pore-forming proteins (17–20). Morphologically, cells undergoing pyroptosis are distinguished by features such as cell swelling, the formation of large bubbles, and eventual rupture resulting in lytic cell death. Notably, patients with KD exhibit higher expression of the NLR family pyrin (PYD) domain-containing 3 (NLRP3) inflammasome, caspase-1, gasdermin D (GSDMD), and downstream proinflammatory cytokines such as IL-1β compared to healthy controls (21).

Here, we outline the pathophysiology of pyroptosis in KD and discuss the potential therapeutic strategies targeting pyroptosis for the treatment of KD.

## Mechanisms of pyroptosis

**Figure 1:**
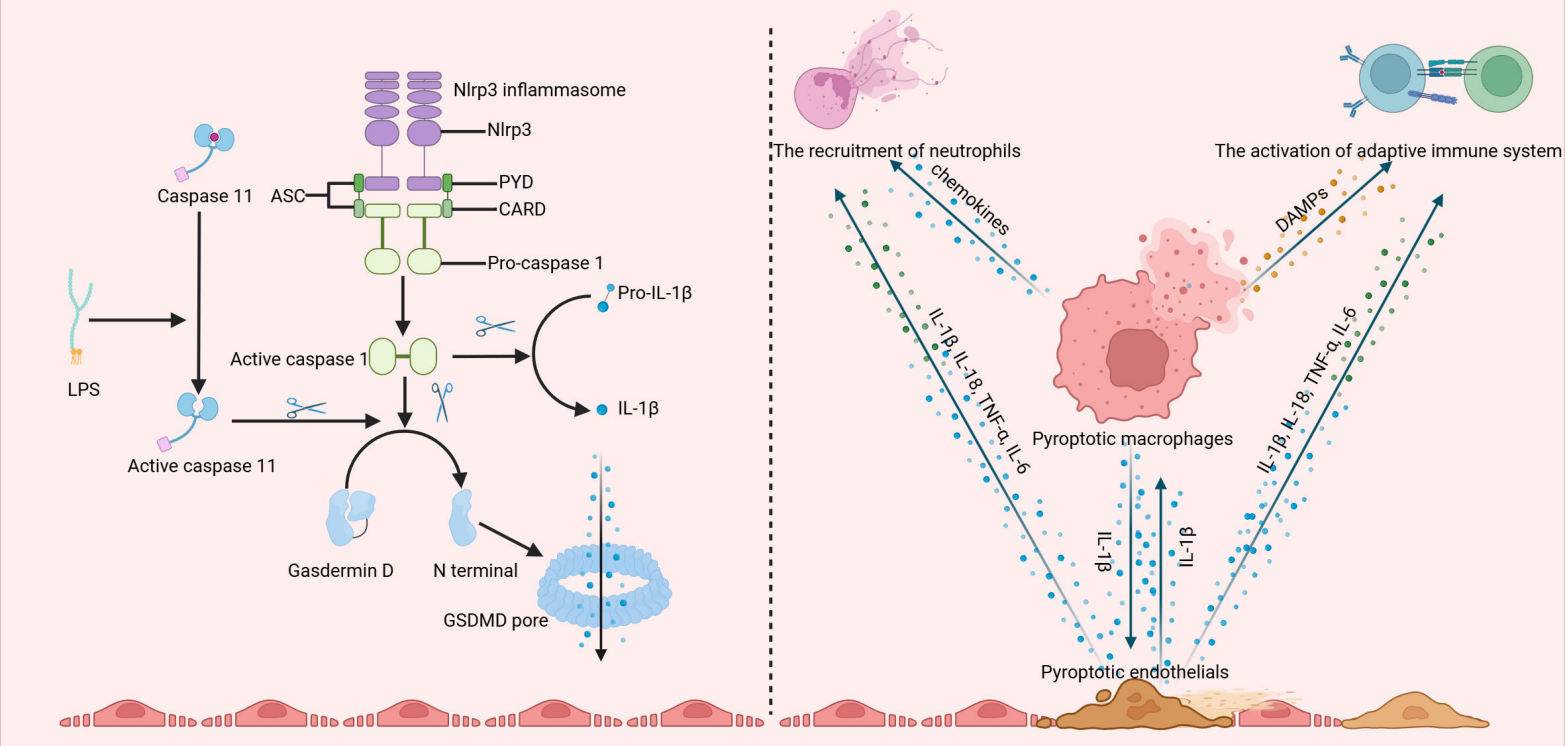
The canonical activation of the inflammasome involves the assembly of the NLRP3 inflammasome, which facilitates the cleavage of procaspase-1 into its active form, caspase-1. Active caspase-1 then cleaves gasdermin D (GSDMD), producing the N-terminal fragment of GSDMD (N-GSDMD). This fragment subsequently inserts into the membrane and oligomerizes, forming pores that ultimately result in cell lysis. Additionally, active caspase-1 is capable of cleaving IL-1β, allowing its release through these pores. Moreover, intracellular lipopolysaccharide (LPS) can activate a non-canonical inflammasome pathway, triggering caspase-11 and leading to the cleavage of GSDMD. Both macrophages and endothelial cells display a pyroptosis-associated phenotype in Kawasaki disease (KD), interacting with one another and contributing to the infiltration of neutrophils and T and B cells. (Created with BioRender.com).

Pyroptosis is a form of inflammatory necrosis initiated by the formation of an inflammasome (17, 18). The canonical activation of the inflammasome relies on NOD-like receptors and non-NOD receptors, which function as sensor proteins. Together with adaptor proteins and the protease caspase-1, the sensor proteins constitute the inflammasome complex, enabling the cleavage of procaspase-1 into active caspase-1. Active caspase-1 then cleaves GSDMD to generate N-terminal GSDMD (N-GSDMD), which then inserts into the membrane and oligomerizes, forming pores that ultimately lead to cell rupture. Additionally, active caspase-1 can also cleave IL-1β and IL-18, releasing them through these pores. Intercellular lipopolysaccharide (LPS) can trigger non-canonical inflammasome-induced pyroptosis, where caspase-11 is activated, leading to the cleavage of GSDMD, IL-1β, and IL-18. The upregulation of GSDMD and IL-1β in the serum of patients with KD indicates a significant role for pyroptosis in its pathogenesis(21, 22). Furthermore, both macrophages and endothelial cells exhibit a pyroptosis-associated pattern in KD, underscoring a crucial role of pyroptosis in inflammation and endothelial cell injury (**Figure 1**).

## Pyroptosis and macrophage-mediated inflammation in KD

Unlike silent apoptosis, pyroptosis is characterized by the abundant release of damage-associated molecular patterns (DAMP) and inflammatory cytokines, such as high mobility group protein B1 (HMGB1) and IL-1β. The inflammation in KD is intricate, involving the activation of immune cells that infiltrate the arterial wall, leading to necrotizing arteritis. Notably, in some severe cases among children, KD has been associated with macrophage activation syndrome, marked by significantly elevated levels of pyroptosis-related proteins, including apoptosis-associated speck-like protein containing CARD (ASC), caspase-1, IL-1β, IL-18, GSDMD, and LDH in their serum (**Table 2**) (23). Many studies have emphasized the crucial role of monocytes and macrophages in the release of cytokines and the resultant destructive inflammation in patients with KD (12, 24). Here, we propose the potential involvement of macrophage pyroptosis in KD, which may contribute to the development of the inflammatory microenvironment.

**Table 2:** Identified targets for pyroptosis in KD.

Target	Therapeutic drug	The source of evidence
NLRP3 (13, 21, 28, 48, 54–56)	MCC590	Animal experiments (28, 33)
Caspase-1 (13, 21, 27, 49, 54, 55)	Ac-FLTD-CMK	*In vitro* experiment (13)
IL-1β (11, 13, 21, 28, 50–52, 54–57)	Anakinra	Clinical studies (39–45)
IL-18 (13, 21, 28, 56)	Unreported	Unreported
ASC (13, 21)	Unreported	Unreported
GSDMD (13, 21, 53, 54)	Necrosulfonamide	*In vitro* experiment (13)

NLRP3, NLR family pyrin domain containing 3; IL-1β, interleukin-1 beta; IL-18, interleukin-18; ASC, apoptosis-associated speck-like protein containing CARD; GSDMD, gasdermin D; Ac-FLTD-CMK, *N*-acetyl-Phe-Leu-Thr-Asp-chloromethylketone.

Macrophages exert a double-sided effect in patients with KD. Macrophages can scavenge pathogenic microorganisms, thereby protecting children from immune deregulation caused by infections (25). However, an excessive presence of pro-inflammatory macrophages can secret cytokines and matrix-degrading enzymes, contributing to inflammation infiltration and destabilization of vascular smooth muscle cells. Researchers have demonstrated the central role of macrophages among the immune cells in KD through single-cell RNA sequencing. They identified that monocytes/macrophages are the primary source of dysregulated immune gene expression in KD, including the upregulation of various cytokines and drug targets, such as IL1B and TNF (26).

Importantly, NLRP3 inflammasome activation in monocytes and macrophages has been observed in both *in vitro* and *in vivo* studies. Liu and colleagues were the first to report the activation of the NLRP3 inflammasome in peripheral blood mononuclear cells (PBMCs) from patients with KD (21). They found that NLRP3 and other pyroptosis-related genes were significantly upregulated in KD, particularly in patients with coronary artery lesions (CALs). Kuo et al. were the first to report the upregulation of pyroptosis-related caspases in leukocytes of KD, including caspase-1, -4, and -5 (27). Intriguingly, the levels of these pyroptosis-related caspases returned to normal after effective treatment with standard-of-care medications. These findings suggest that pyroptosis in peripheral leukocytes plays a critical role in the inflammatory pathogenesis of KD.

However, the susceptibility mechanisms of the activation of the NLRP3 inflammasome in KD remain unclear. Inositol-triphosphate 3-kinase C (ITPKC), a widely known KD-associated gene, has been shown to regulate NLRP3 by modulating intracellular calcium levels (28). Animal models based on *Itpkc*-deficient mice further support this perspective. Recently, Jin et al. revealed significant crosstalk between neutrophil extracellular traps (NETs) and PBMC pyroptosis (14). They found that elevated levels of NETs in KD patients were sufficient to induce the activation of the NLRP3 inflammasome via a ROS-dependent signaling pathway, resulting in the pyroptosis of PBMCs, particularly monocytes, and the release of inflammatory mediators such as IL-1β. These findings indicate a close correlation between pyroptosis and the inflammatory factors derived from monocytes and macrophages.

**Figure 2:**
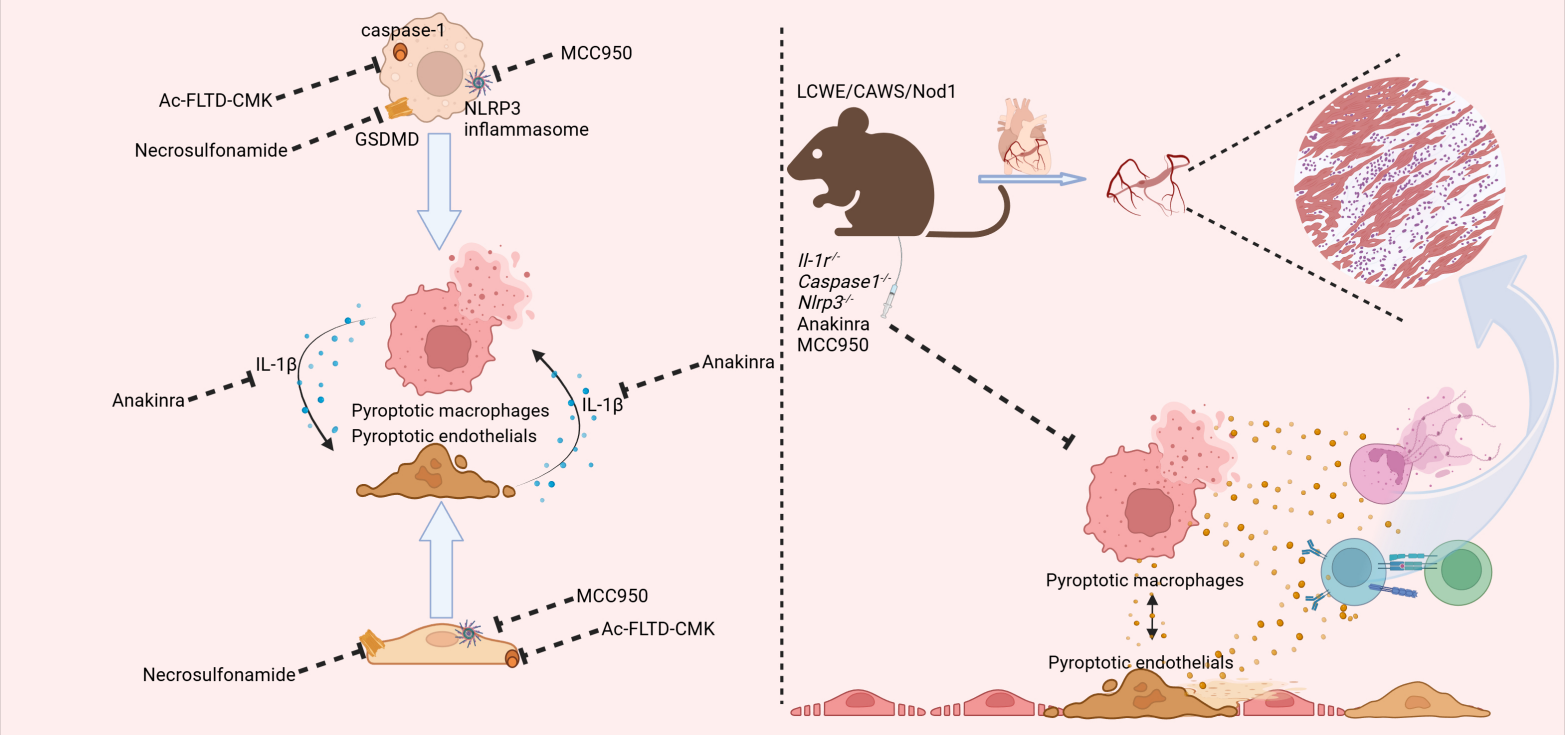
The findings from both *in vivo* and *in vitro* studies have converged to highlight the pivotal role of pyroptosis-related pathways in the pathogenesis of KD. In the realm of *in vitro* research, the employment of the caspase1 inhibitor *N*-acetyl-Phe-Leu-Thr-Asp-chloromethylketone (Ac-FLTD-CMK), the gasdermin D (GSDMD) inhibitor necrosulfamide, and the NLR family pyrin domain containing 3 (NLRP3) inhibitor MCC950 has collectively demonstrated the ability to suppress pyroptosis within macrophages and endothelial cells. This suppression, in turn, serves to temper the subsequent inflammatory responses. Additionally, the interleukin-1 receptor antagonist anakinra has been shown to inhibit the downstream effects of interleukin-1 beta (IL-1β), thereby dampening the amplification of the inflammatory cascade between macrophages and endothelial cells. In the context of *in vivo* experimentation, mice deficient in *Il-1r*, *Caspase-1*, and *Nlrp3* exhibited a marked attenuation in the progression of coronary inflammation, as observed in KD mice. Moreover, both anakinra and MCC950 were found to exert similar protective effects, further corroborating the significance of targeting these pathways in the treatment of KD. (Created with BioRender.com).

*In vivo* studies have demonstrated that pyroptosis plays a significant role in macrophage-mediated inflammation (**Figure 2**). Currently, there are three main types of animal models for KD, including *Lactobacillus casei* cell wall extract (LCWE), *Candida albicans* water-soluble fraction (CAWS), and nucleotide-binding oligomerization domain containing 1 (Nod1) ligand-induced mice (10). Among these, the LCWE-induced animal models are regarded to have pathological changes that most closely resemble those seen in children with KD (29). Moreover, both the NLRP3 inflammasome and the pyroptosis pathway are crucial in the progression of coronary lesions. In LCWE-induced animal models, increased macrophage-derived IL-1β maturation and secretion have been observed (30).

Additionally, these biological processes were found to be highly dependent on the activation of the NLRP3 inflammasome and caspase-1 in macrophages. Both caspase-1-deficient and IL-1 receptor-deficient mice exhibited reduced coronary lesions in response to LCWE, further suggesting the potential role of macrophage pyroptosis in KD (12, 31). Subsequent investigation revealed that deficiency in caspase-1 and NLRP3 in macrophages could inhibit LCWE-induced abdominal aortic aneurysm formation. Furthermore, in addition to IL-1β, IL-1α also contributed to the pathogenesis of KD, highlighting the significance of macrophage pyroptosis and its crucial role in KD. Notably, pyroptosis-derived IL-1β also played an essential role in the development of coronary injury in a CAWS-induced KD mouse model (32). Takahashi and colleagues found that CAWS induced the expression of Dectin-2 in macrophages, which activates the NF-κB pathway, leading to the assembly of the inflammasome and the subsequent cleavage of caspase-1 (33). Both IL-1β and NLRP3 deficiency could protect mice from the induction of vasculitis in these models. In the Nod1 ligand mouse model, a significant infiltration of macrophages into the coronary arteries was observed (34, 35). Furthermore, specific depletion of macrophages significantly attenuated Nod1 ligand-induced vasculitis. Surprisingly, the concentration of circulating IL-1β was significantly higher and correlated with macrophage-mediated inflammation (36).

In all three models, the activation of pyroptosis-related pathways in macrophages played a crucial role. Nevertheless, few research studies have investigated the role of GSDMD, the executor of pyroptosis, in these animal models. More in-depth investigation on macrophage pyroptosis is warranted in the future.

## Pyroptosis and endothelial cell injuries in KD

The injury of endothelial cells is one of the hallmark pathophysiological processes in KD. In addition to the previously discussed relationship between pyroptosis and macrophage-mediated inflammation, pyroptosis occurring in endothelial cells can directly lead to their destruction, contributing to the formation of coronary aneurysm. Jia et al. were the first to demonstrate that KD serum induces macrophages to trigger pyroptosis in human umbilical vein endothelial cells (HUVECs), featured by the activation of the NLRP3 inflammasome and the cleavage of GSDMD and IL-1β (13). Notably, GSDMD-related pyroptosis inhibitors such as necrosulfonamide significantly mitigated the release of IL-1β induced by endothelial cell pyroptosis.

Furthermore, subsequent experiments confirmed that the HMGB1/RAGE/cathepsin B signaling pathway is involved in activating the NLRP3 inflammasome in endothelial cells, leading to pyroptosis. These *in vitro* studies clearly demonstrated that endothelial cells can undergo pyroptosis in the context of KD. Subsequently, the authors found that inhibiting the NLRP3 inflammasome attenuated the pyroptosis of endothelial cells in CAWS-induced animal models. Thus, endothelial pyroptosis may help explain the endothelial injury observed in coronary arteries in KD. Furthermore, in the Nod1 ligand mouse model, delivery of the Nod1 ligand specifically activates vascular cells, such as endothelial cells, leading to elevated production of IL-1β. This, in turn, results in the recruitment of inflammatory cells and the expansion of the inflammatory cascade, suggesting a close correlation between pyroptosis and endothelial cells (36, 37).

In fact, animal models with endothelial cell conditional knockout of pyroptosis-related proteins are needed to accurately illustrate the role of pyroptosis in endothelial cell injuries in KD. Moreover, direct immunohistochemical staining of the pyroptosis pathway in endothelial cells from patients with KD is also needed. Therefore, further improvements in this area of research are necessary.

## Potential interventions targeted pyroptosis

Although the use of high-dose IVIG together with aspirin has significantly reduced the risk of developing coronary artery lesions in patients with KD, approximately 20% of patients still exhibit a poor response to the above treatment and remain at higher risk for coronary artery complications (9). Given the crucial role of pyroptosis in the progression of KD, targeting this process may represent a promising treatment strategy, particularly for those with IVIG-resistant KD (**Table 2**).

IL-1β and IL-18 are important inflammatory factors in the pyroptosis pathway. Many studies have demonstrated that blocking the IL-1β pathway may serve as a valid therapeutic option for KD. Insights from mouse models suggested that IL-1α, IL-1β, and their receptors are essential for the development of KD-associated vasculitis (12, 30, 31, 38). Anakinra, a recombinant human interleukin-1 receptor (IL-1R) antagonist, has previously been used for the treatment of macrophage activation syndrome. Notably, a growing body of case reports highlights the successful use of anakinra in patients with IVIG-resistant KD (39–42). In a study conducted by Isabelle et al., 11 patients with KD who did not respond to immunoglobulin were treated with anakinra, resulting in significant improvements in their body temperature, systemic inflammatory markers, and coronary artery dilation (43). An open-label Phase IIa clinical trial further evaluated the safety of anakinra in patients with IVIG-resistant KD (44). The results from a Phase I/IIa trial by Jin et al. also confirmed that anakinra is safe for clinical use in both infants and children with KD and coronary artery aneurysms (45). Based on these findings, we believe that anakinra may have therapeutic effects in patients with refractory KD, particularly in severe complications such as Kawasaki shock syndrome. It could also help alleviate coronary artery dilation and aneurysms or prevent their deterioration. As a result, anakinra is currently being evaluated in clinical trials, and future randomized controlled trials with longer durations and larger sample sizes are necessary to verify its safety and effectiveness. While elevated levels of IL-18 in the serum of patients with KD have been observed, no animal or clinical studies targeting IL-18 have been performed (46). Recombinant human IL-18 binding protein, a natural IL-18 inhibitor, has shown promise in many inflammation-associated diseases and could potentially be beneficial in the treatment of KD (47).

The activation of NLRP3 is a pivotal event in the pyroptosis pathway. Theoretically, directly targeting NLRP3 with small molecule inhibitors could offer additional therapeutic benefits for KD by blocking downstream effects of pyroptosis, including the release of cellular contents and the production of IL-1β and IL-18. For example, MCC590, an inhibitor of NLRP3, has been shown to significantly alleviate the coronary artery inflammation induced by CAWS (33). Nevertheless, it is important to note that all studies to date have been conducted in animal models, with no clinical trial yet performed. In addition to the NLRP3 inflammasome, other components of the pyroptosis pathway warrant attention. Mice with a caspase-1 deficiency were protected from LCWE-induced coronary artery lesions. *N*-acetyl-Phe-Leu-Thr-Asp-chloromethylketone (Ac-FLTD-CMK), a carefully designed selective inhibitor of caspase-1, -4, -5, and -11, has been proven to effectively alleviate pyroptosis in endothelial cells *in vitro* (13). However, there is a paucity of research examining the effects of targeting caspases on KD in *in vivo* studies. Similarly, necrosulfonamide, a small molecular inhibitor of GSDMD, has demonstrated the ability to attenuate cell death and reduce IL-1β release in *in vitro* studies (13). Thus, more in-depth studies are needed to systematically explore the effects of targeting caspases and GSDMD *in vivo*, progressing from animal models to clinical trials.

## Conclusion

Pyroptosis is a distinct inflammatory death pattern, characterized by the release of inflammatory cytokines and rupture of membranes. Existing literature indicates that pyroptosis in various cell types, including macrophages and endothelial cells, plays a significant role in the progression of KD. In this review, we elucidate the molecular mechanisms underlying pyroptosis in KD and present new insights into pyroptosis-targeting therapeutic strategies. Our goal is to enhance the efficacy of current KD therapies, particularly for patients who are resistant to intravenous immunoglobulin therapy.
